# The genome sequence of the snout, *Hypena proboscidalis* (Linnaeus, 1758)

**DOI:** 10.12688/wellcomeopenres.17189.1

**Published:** 2021-09-15

**Authors:** Douglas Boyes, Peter W.H. Holland, University of Oxford and Wytham Woods Genome Acquisition Lab, Darwin Tree of Life Barcoding collective, Wellcome Sanger Institute Tree of Life programme, Wellcome Sanger Institute Scientific Operations: DNA Pipelines collective, Tree of Life Core Informatics collective, Darwin Tree of Life Consortium

**Affiliations:** 1 UK Centre for Ecology & Hydrology, Wallingford, Oxfordshire, OX10 8BB, UK; 2 Department of Zoology, University of Oxford, Oxford, OX1 3SZ, UK

**Keywords:** Hypena proboscidalis, the snout, genome sequence, chromosomal

## Abstract

We present a genome assembly from an individual female *Hypena proboscidalis *(the snout; Arthropoda; Insecta; Lepidoptera; Erebidae). The genome sequence is 637 megabases in span. The majority of the assembly is scaffolded into 31 chromosomal pseudomolecules, with the Z sex chromosome assembled.

## Species taxonomy

Eukaryota; Metazoa; Ecdysozoa; Arthropoda; Hexapoda; Insecta; Pterygota; Neoptera; Endopterygota; Lepidoptera; Glossata; Ditrysia; Noctuoidea; Erebidae; Hypeninae; Hypena; *Hypena proboscidalis* Linnaeus 1758 (NCBI:txid753189).

## Introduction

Caterpillars of *Hypena proboscidalis* (the snout) are specialised herbivores of nettle plants; the common and binomial names reference the prominent labial palps of the adult. The genome of *H. proboscidalis* was sequenced as part of the Darwin Tree of Life Project, a collaborative effort to sequence all of the named eukaryotic species in the Atlantic Archipelago of Britain and Ireland. Here we present a chromosomally complete genome sequence for *H. proboscidalis*, based on one female specimen from Wytham Woods, Oxfordshire, UK.

## Genome sequence report

**Figure 1.**
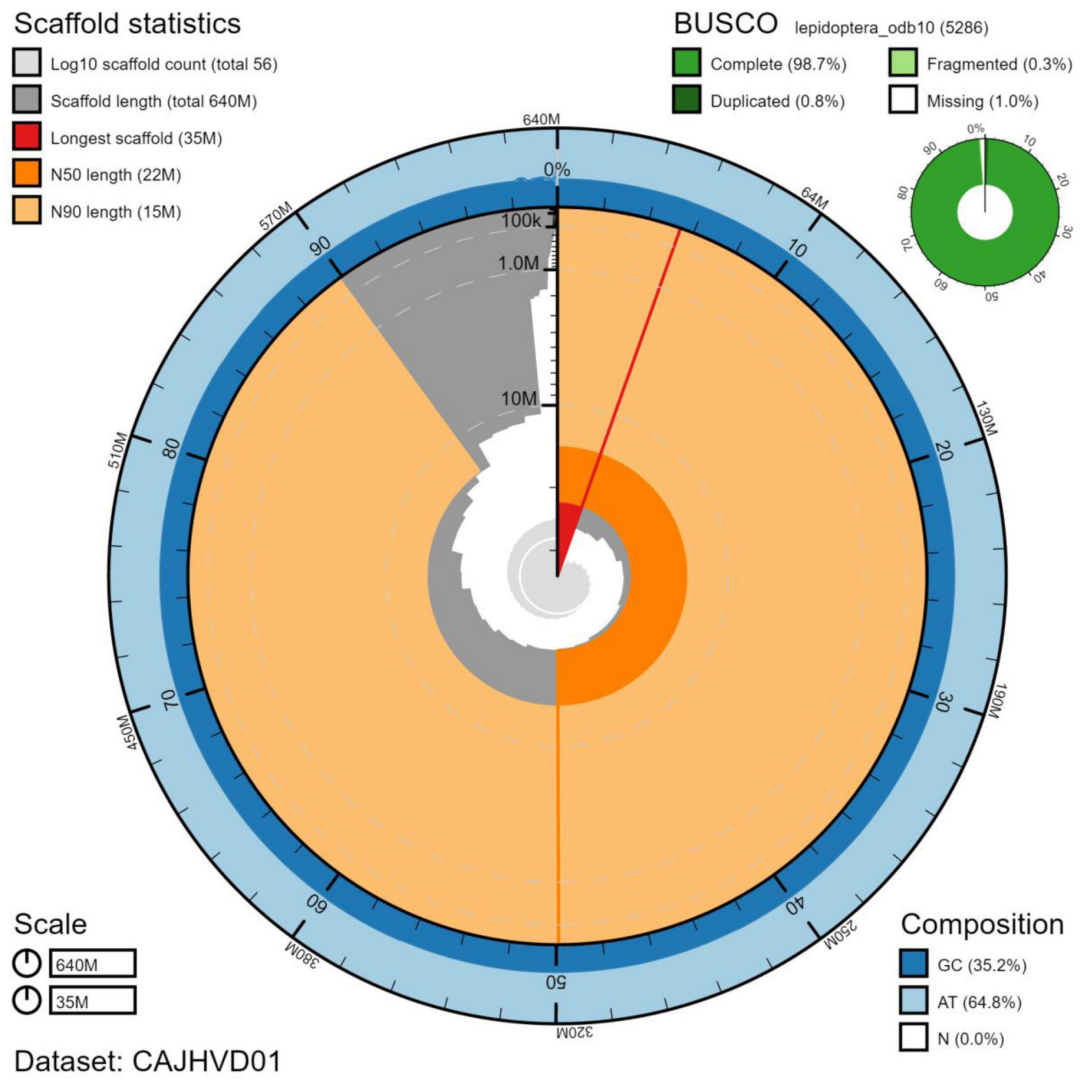
Genome assembly of *Hypena proboscidalis*, ilHypProb1.1: metrics. The BlobToolKit Snailplot shows N50 metrics and BUSCO gene completeness. An interactive version of this figure is available at https://blobtoolkit.genomehubs.org/view/ilHypProb1.1/dataset/CAJHVD01/snail.

The genome was sequenced from a single female *H. proboscidalis* collected from Wytham Woods, Oxfordshire, UK (latitude 51.772, longitude -1.338). A total of 23-fold coverage in Pacific Biosciences single-molecule long reads (N50 18 kb) and 55-fold coverage in 10X Genomics read clouds were generated. Primary assembly contigs were scaffolded with chromosome conformation Hi-C data. Manual assembly curation corrected 34 missing/misjoins and removed 14 haplotypic duplications, reducing the assembly length by 2.17% and the scaffold number by 28.57%, and increasing the scaffold N50 by 14.21%. The final assembly has a total length of 637 Mb in 56 sequence scaffolds with a scaffold N50 of 22 Mb ( Table 1). The majority, 98.3%, of assembly sequence was assigned to 31 chromosomal-level scaffolds, representing 30 autosomes (numbered by sequence length), and the Z sex chromosome ( Figure 1– Figure 4; Table 2). The assumed sex chromosome karyotype is Z0. The assembly has a BUSCO v5.1.2 ( Simão *et al*., 2015) completeness of 98.7% using the lepidoptera_odb10 reference set. While not fully phased, the assembly deposited is of one haplotype. Contigs corresponding to the second haplotype have also been deposited.

**Table 1.** Genome data for *Hypena proboscidalis*, ilHypProb1.1.

*Project accession data*	
Assembly identifier	ilHypProb1.1
Species	*Hypena proboscidalis*
Specimen	ilHypProb1
NCBI taxonomy ID	NCBI:txid753189
BioProject	PRJEB42129
BioSample ID	SAMEA7520188
Isolate information	Female, head/abdomen/thorax
*Raw data accessions*	
PacificBiosciences SEQUEL II	ERR6406200
10X Genomics Illumina	ERR6002650, ERR6002651, ERR6003040, ERR6003041
Hi-C Illumina	ERR6002652
Illumina PolyA RNA-Seq	ERR6002653
*Genome assembly*	
Assembly accession	GCA_905147285.1
*Accession of alternate haplotype*	GCA_905147305.1
Span (Mb)	637
Number of contigs	86
Contig N50 length (Mb)	21
Number of scaffolds	56
Scaffold N50 length (Mb)	22
Longest scaffold (Mb)	26
BUSCO * genome score	C:98.7%[S:98.0%,D:0.8%],F:0.3 %,M:1.0%,n:5286

*BUSCO scores based on the lepidoptera_odb10 BUSCO set using v5.1.2. C= complete [S= single copy, D=duplicated], F=fragmented, M=missing, n=number of orthologues in comparison. A full set of BUSCO scores is available at https://blobtoolkit.genomehubs.org/view/ilHypProb1.1/dataset/CAJHVD01/busco.

**Figure 2.**
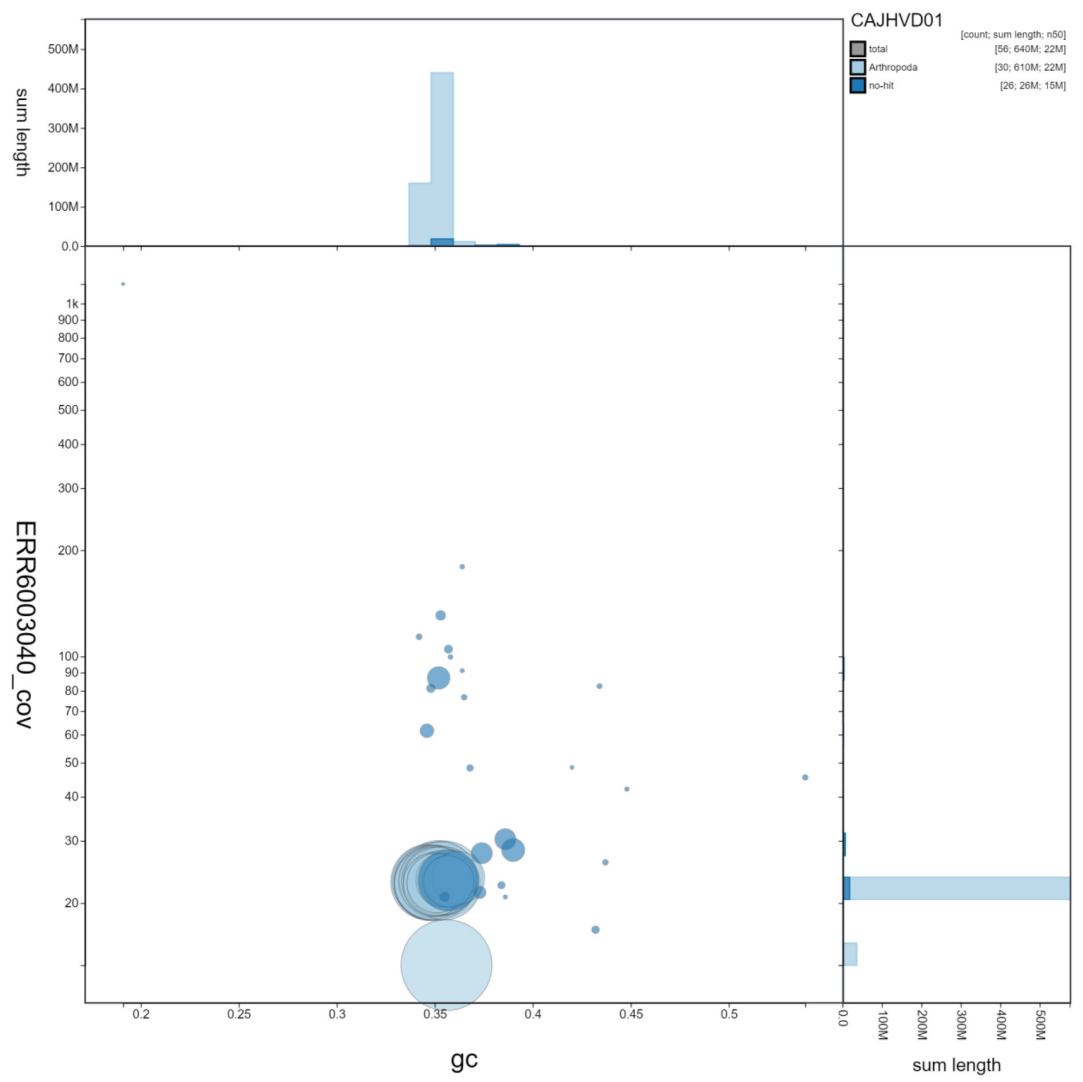
Genome assembly of *Hypena proboscidalis*, ilHypProb1.1: GC coverage. BlobToolKit GC-coverage plot. Scaffolds are coloured by phylum. Circles are sized in proportion to scaffold length. Histograms show the distribution of scaffold length sum along each axis. An interactive version of this figure is available at https://blobtoolkit.genomehubs.org/view/ilHypProb1.1/dataset/CAJHVD01/blob.

**Figure 3.**
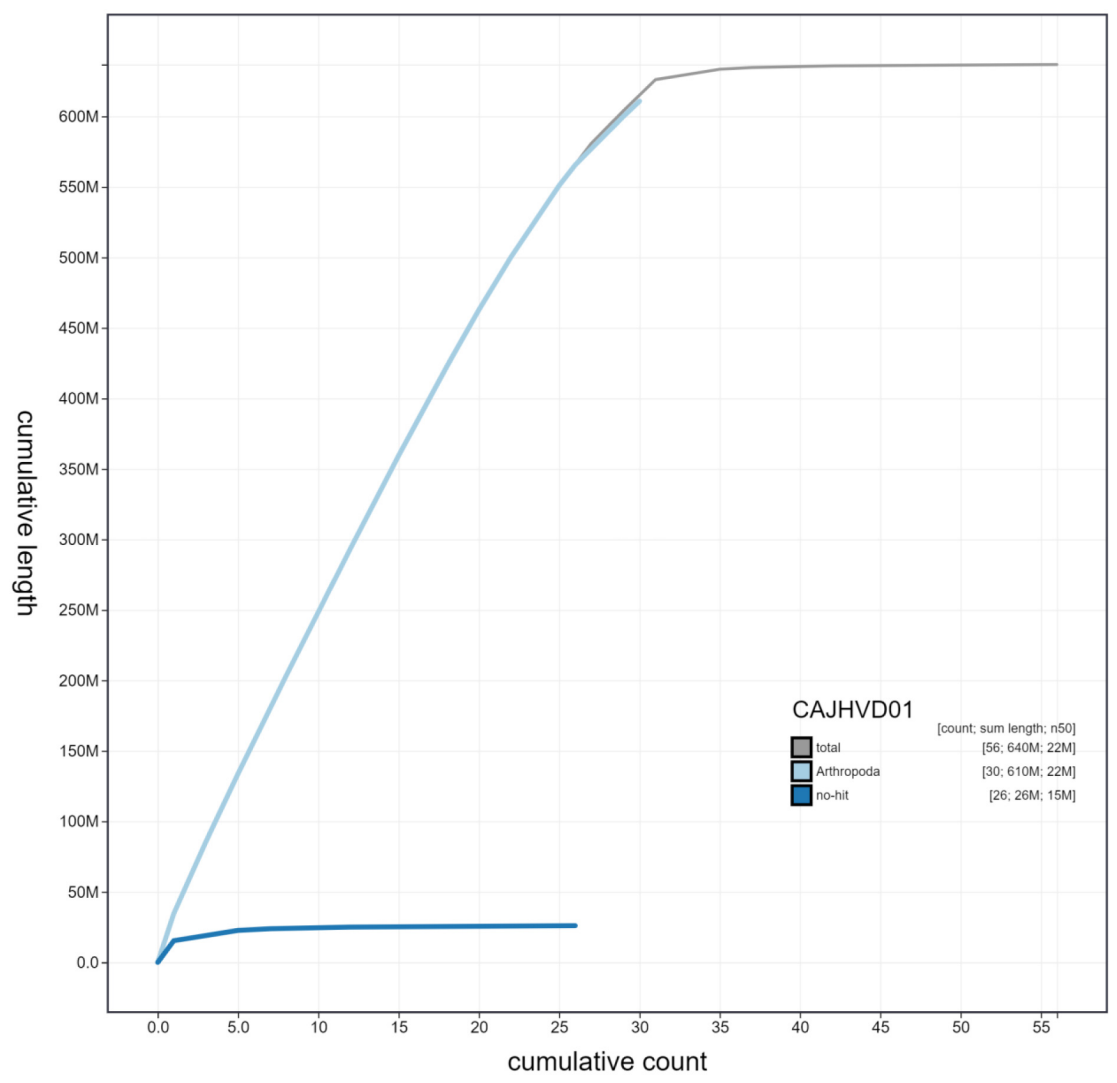
Genome assembly of *Hypena proboscidalis*, ilHypProb1.1: cumulative sequence. BlobToolKit cumulative sequence plot. The grey line shows cumulative length for all chromosomes. Coloured lines show cumulative lengths of chromosomes assigned to each phylum using the buscogenes taxrule. An interactive version of this figure is available at https://blobtoolkit.genomehubs.org/view/ilHypProb1.1/dataset/CAJHVD01/cumulative.

**Figure 4.**
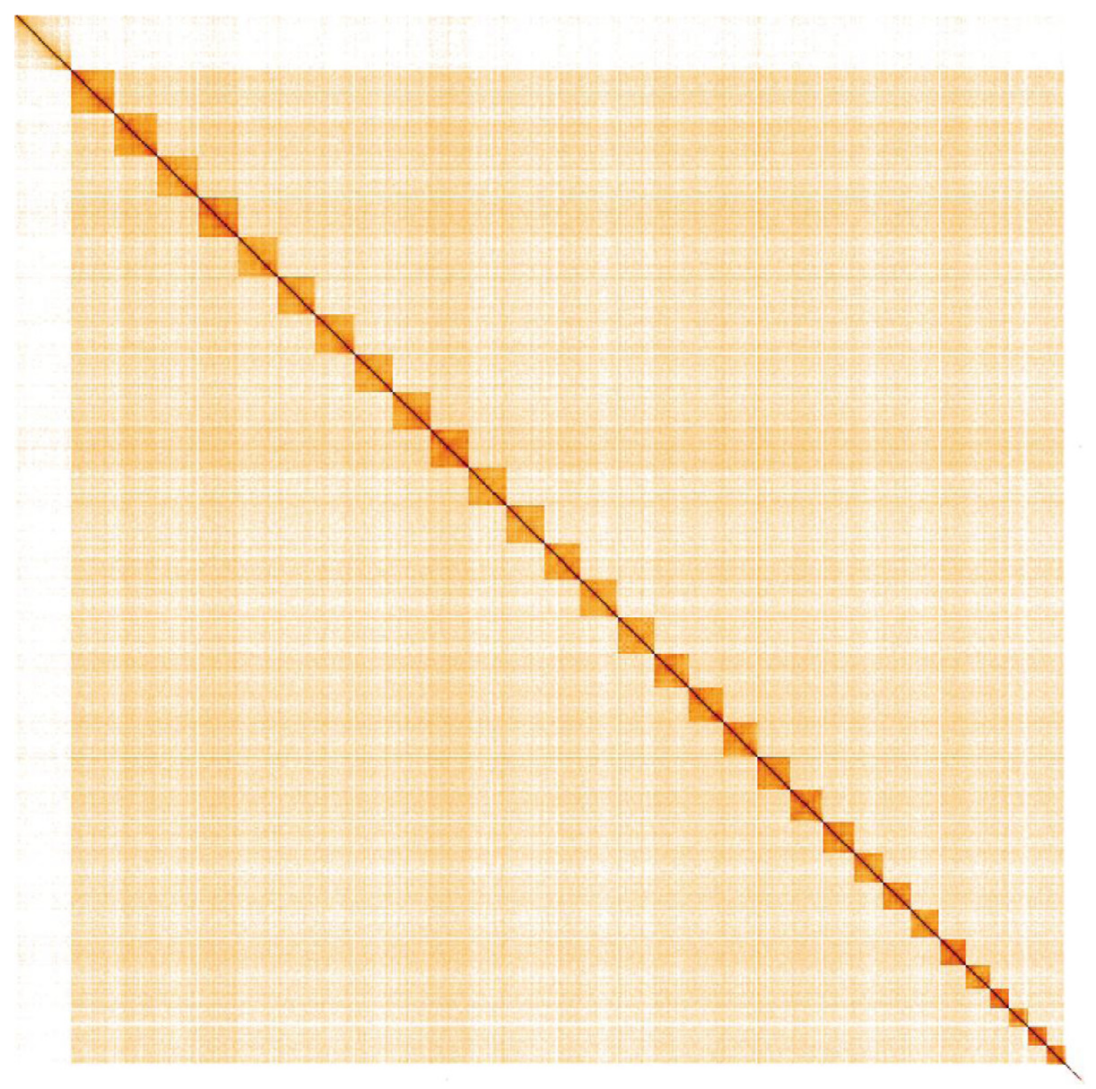
Genome assembly of *Hypena proboscidalis*, ilHypProb1.1: Hi-C contact map. Hi-C contact map of the ilHypProb1.1 assembly, visualised in HiGlass.

**Table 2.** Chromosomal pseudomolecules in the genome assembly of *Hypena proboscidalis*, ilHypProb1.1.

INSDC accession	Chromosome	Size (Mb)	GC%
LR990127.1	1	25.59	35.1
LR990128.1	2	25.33	35.2
LR990129.1	3	24.21	34.7
LR990130.1	4	24.04	35.5
LR990131.1	5	23.26	34.8
LR990132.1	6	23.15	34.8
LR990133.1	7	23.08	34.9
LR990134.1	8	22.66	34.8
LR990135.1	9	22.65	35
LR990136.1	10	22.64	35.3
LR990137.1	11	22.30	34.7
LR990138.1	12	22.17	34.8
LR990139.1	13	21.97	35
LR990140.1	14	21.88	34.8
LR990141.1	15	21.39	35.5
LR990142.1	16	21.18	35
LR990143.1	17	20.50	35.1
LR990144.1	18	20.12	35.2
LR990145.1	19	19.97	35.4
LR990146.1	20	18.83	35.3
LR990147.1	21	18.67	35.1
LR990148.1	22	17.10	35.2
LR990149.1	23	17.06	35.5
LR990150.1	24	16.60	35
LR990151.1	25	15.36	35.6
LR990152.1	26	14.56	35.1
LR990153.1	27	11.71	35.4
LR990154.1	28	11.47	35.8
LR990155.1	29	11.35	36.2
LR990156.1	30	10.71	35.7
LR990126.1	Z	34.62	35.6
LR990157.1	MT	0.02	19.3
-	Unplaced	10.64	37.4

## Methods

A female *H. proboscidalis*, ilHypProb1, and a second specimen of unknown sex, ilHypProb2, were collected from Wytham Woods, Oxfordshire, UK (latitude 51.772, longitude -1.338) by Douglas Boyes, University of Oxford using a light trap. The specimens were snap-frozen in dry ice using a CoolRack before transferring to the Wellcome Sanger Institute (WSI).

DNA was extracted from head and thorax tissue of ilHypProb1 by the Scientific Operations core at the WSI using the Qiagen MagAttract HMW DNA kit, according to the manufacturer’s instructions. RNA was extracted from ilHypProb2 in the Tree of Life Laboratory at the WSI using TRIzol (Invitrogen), according to the manufacturer’s instructions. RNA was then eluted in 50 μl RNAse-free water and its concentration RNA assessed using a Nanodrop spectrophotometer and Qubit Fluorometer using the Qubit RNA Broad-Range (BR) Assay kit. Analysis of the integrity of the RNA was done using Agilent RNA 6000 Pico Kit and Eukaryotic Total RNA assay.

Pacific Biosciences HiFi circular consensus and 10X Genomics read cloud DNA sequencing libraries, in addition to PolyA RNA-Seq libraries, were constructed according to the manufacturers’ instructions. Sequencing was performed by the Scientific Operations core at the WSI on Pacific Biosciences SEQUEL II (HiFi) Illumina HiSeq X (10X) and Illumina HiSeq 4000 (RNA-Seq) instruments. Hi-C data were generated from abdomen tissue using the Qiagen EpiTect Hi-C kit and sequenced on HiSeq X.

Assembly was carried out with Hifiasm ( Cheng *et al*., 2021); haplotypic duplication was identified and removed with purge_dups ( Guan *et al*., 2020). The assembly was polished with the 10X Genomics Illumina data by aligning to the assembly with longranger align, calling variants with freebayes ( Garrison & Marth, 2012). One round of the Illumina polishing was applied. Scaffolding with Hi-C data ( Rao *et al*., 2014) was carried out with SALSA2 ( Ghurye *et al*., 2019). The assembly was checked for contamination and corrected using the gEVAL system ( Chow *et al*., 2016) as described previously ( Howe *et al*., 2021). Manual curation was performed using gEVAL, HiGlass ( Kerpedjiev *et al*., 2018) and Pretext. The mitochondrial genome was assembled using MitoHiFi ( Uliano-Silva *et al*., 2021). The genome was analysed and BUSCO scores generated within the BlobToolKit environment ( Challis *et al*., 2020). Table 3 contains a list of all software tool versions used, where appropriate.

**Table 3.** Software tools used.

Software tool	Version	Source
Hifiasm	0.12	Cheng *et al*., 2021
purge_dups	1.2.3	Guan *et al*., 2020
SALSA2	2.2	Ghurye *et al*., 2019
longranger align	2.2.2	https://support.10xgenomics.com/genome-exome/ software/pipelines/latest/advanced/other-pipelines
freebayes	1.3.1-17-gaa2ace8	Garrison & Marth, 2012
MitoHiFi	1.0	Uliano-Silva *et al*., 2021
gEVAL	N/A	Chow *et al*., 2016
HiGlass	1.11.6	Kerpedjiev *et al*., 2018
PretextView	0.1.x	https://github.com/wtsi-hpag/PretextView
BlobToolKit	2.6.2	Challis *et al*., 2020

The materials that have contributed to this genome note have been supplied by a Darwin Tree of Life Partner. The submission of materials by a Darwin Tree of Life Partner is subject to the Darwin Tree of Life Project Sampling Code of Practice. By agreeing with and signing up to the Sampling Code of Practice, the Darwin Tree of Life Partner agrees they will meet the legal and ethical requirements and standards set out within this document in respect of all samples acquired for, and supplied to, the Darwin Tree of Life Project. Each transfer of samples is further undertaken according to a Research Collaboration Agreement or Material Transfer Agreement entered into by the Darwin Tree of Life Partner, Genome Research Limited (operating as the WSI), and in some circumstances other Darwin Tree of Life collaborators.

## Data availability

European Nucleotide Archive: Hypena proboscidalis (the snout). Accession number PRJEB42129: https://identifiers.org/ena.embl:PRJEB42129.

The genome sequence is released openly for reuse. The *H. proboscidalis* genome sequencing initiative is part of the Darwin Tree of Life (DToL) project. All raw sequence data and the assembly have been deposited in INSDC databases. The genome will be annotated using the RNA-Seq data and presented through the Ensembl pipeline at the European Bioinformatics Institute. Raw data and assembly accession identifiers are reported in Table 1.
