# Supplementary material for: The genome sequence of the snout, Hypena proboscidalis (Linnaeus, 1758)
Source: Wellcome Open Res. 2021 Sep 15;6:236. [Version 1] doi: 10.12688/wellcomeopenres.17189.1 (PMC9971693; doi:10.12688/wellcomeopenres.17189.1)
Supplement: Peer review history

# Peer review: Reviewer response for version 1

**DOI:** 10.21956/wellcomeopenres.18992.r54363

**Published:** 2023-02-10

**Authors:** Kuppusamy Sivasankaran

**Affiliations:** 1 Loyola Collège, Chennai, Tamilnadu, India

**Review report**

 I appreciate the authors for assembling the whole genome sequence of Snout *Hypena proboscidalis*. The appropriate software, genome assembling process, and whole genome sequencing technologies were used.

**The few comments on the manuscripts.  **

- Total sequence lengths (636,786,269 bp) were not mentioned in the text.
- Non-nuclear genome size and Scaffold count not mentioned in the text.
- How many nuclear genes were established in the assembling of the entire genome? The text makes no mention of the nuclear gene numbers.
- The maximum and minimum contig length was not indicated. Total contigs length was not indicated (18,834,925 bp).
- Gene recovery statistics can be given in the text.
- Overall, the manuscript is written well and it can be accepted for indexing.

Are sufficient details of methods and materials provided to allow replication by others?

Yes

Is the rationale for creating the dataset(s) clearly described?

Yes

Are the datasets clearly presented in a useable and accessible format?

Yes

Are the protocols appropriate and is the work technically sound?

Yes

Reviewer Expertise:

Phylogenetic analysis of the superfamily Noctuoidea moths using mitochondrial genome sequence

I confirm that I have read this submission and believe that I have an appropriate level of expertise to confirm that it is of an acceptable scientific standard.

# Peer review: Reviewer response for version 1

**DOI:** 10.21956/wellcomeopenres.18992.r54390

**Published:** 2023-02-08

**Authors:** Zdenek F. Fric

**Affiliations:** 1 Institute of Entomology,, Branisovska, Czech Republic

The paper introduces a newly published genome of another British moth, continuing in the Darwin Tree of Life Project, where the authors try to sequence all known species occurring in British islands. The aim is clear, the methodology is identical to all previous projects of the DTLP and the exact pipeline commands are in the same way obscured. However, the initiative is an important step in knowledge and the resulting DTLP sequences are a very useful tool for other genomic projects as they are easy to use as a reference genome for all other analyses.

Are sufficient details of methods and materials provided to allow replication by others?

Yes

Is the rationale for creating the dataset(s) clearly described?

Yes

Are the datasets clearly presented in a useable and accessible format?

Yes

Are the protocols appropriate and is the work technically sound?

Yes

Reviewer Expertise:

Lepidoptera phylogeny and evolution

I confirm that I have read this submission and believe that I have an appropriate level of expertise to confirm that it is of an acceptable scientific standard.

# Peer review: Reviewer response for version 1

**DOI:** 10.21956/wellcomeopenres.18992.r45888

**Published:** 2021-09-30

**Authors:** Angela McGaughran

**Affiliations:** 1 Te Aka Mātuatua/School of Science, University of Waikato, Hamilton, New Zealand

The rationale for the study is not particularly defined - there is a very brief introduction to the species and mention that the sequencing is part of the DTLP, but some more information could be provided about the species. E.g., is it a pest, is it endangered, is it important in ecosystem function, etc.  This would help place the study a bit better contextually.

 The Methods section outlines all of the software used and version numbers are provided in Table 3. But there is no detail of the actual commands used, so the study cannot be replicated. Provision of the bioinformatic coding in Supplementary material would address this.

 The figures are attractive to look at, but the legends are very brief and therefore the figures are not easy to interpret. For example, in Fig 4 the axes are not labelled; in Fig 1, the relevant percentages or other values seem to all be stated in the associated keys, so, though the figure looks great, it's not clear what it's adding. There is also no description of what the figures mean in the text.

Are sufficient details of methods and materials provided to allow replication by others?

Partly

Is the rationale for creating the dataset(s) clearly described?

Partly

Are the datasets clearly presented in a useable and accessible format?

Partly

Are the protocols appropriate and is the work technically sound?

Yes

Reviewer Expertise:

Genomics, population genomics.

I confirm that I have read this submission and believe that I have an appropriate level of expertise to confirm that it is of an acceptable scientific standard, however I have significant reservations, as outlined above.
